# The Texas Medical College

**Published:** 1901-07

**Authors:** 


### THE TEXAS MEDICAL COLLEGE.

The graduation exercises of the Medical Department of the University of Texas, at Galveston, were held on the evening of June 15, in Cathedral Hall in Galveston. The usual program was carried out, President Prather conferring the degree of Doctor of Medicine upon a class of six, that of Graduate of Pharmacy upon a class of fourteen, and certificates of proficiency as trained nurses upon a class of eight.

During the past session there have been enrolled in the department in all one hundred and ninety-one students, distributed as follows: Special, one; Fourth Year Medicine, six; Third Year Medicine, sixteen; Second Year Medicine, forty; First Year Medicine, fifty-five; Second Year Pharmacy, sixteen; First Year Pharmacy, thirty-six; Second Year Nursing, eight; First Year Nursing, thirteen. It is a matter of note that, while there has been a considerable diminution in size of the classes in medicine, those in pharmacy have this year been larger than ever before, a fact explicable by the excellence of instruction in this school and the little competition in the teaching of this branch in the South.

The small size of the medical classes is due to a number of causes. The prospects for an unusually large entering class were good prior to the storm of last September, and doubtless this disaster diverted a large number from the school at Galveston. It is actually known to have prevented the return of a large number of those already in attendance.

The graduating class in medicine is the first which has regularly pursued the entire four year course of instruction and originally numbered sixty members. It entered during the yellow fever excitement of 1897; and this, with the lengthening of the course, was regarded as the cause of its small membership. The following year much the same conditions prevailed, and a similarly small class was matriculated. In 1899, however, the entering class numbered over ninety students, and in 1900 it was confidently predicted that the first year class would reach one hundred and twenty-five. It is to be hoped that, with the buildings again placed in good state and normal conditions prevailing in Galveston, the opening class in medicine will once more assume encouraging size. The school is to be congratulated upon the fact that even in the stress of the past few years no tendency to lowering of standard has been permitted, the annual loss of each class by reason of failures showing clearly the maintenance of full requirements for advance.

At their meeting in Galveston at the time of commencement, the Regents of the University entered into contract for the final repairs to the buildings of the school, and it may be said that in a number of respects these repairs, while costing less than the original cost of construction, will place the plant in a more efficient condition than ever. From gifts to the school and hospital there have accrued, too, several new features, most important of which are the new brick negro wards to replace the old and disreputable quarters for negroes in Sealy Hospital. It should be recorded, too, that the repairs to Sealy Hospital, thorough in every way, were made without cost to the State, Mr. John Sealy and his sister, Mrs. Waverly Smith, bearing the entire expense.

A number of changes in the arrangement of work by members of the teaching staff were also authorized by the Board of Regents. Dr. H. P. Cooke resigned his positions as dean of the faculty, member of the board of hospital managers and professor of pediatrics. Dr. A. J. Smith was appointed to the deanship, Dr. J. E. Thompson to be member of the hospital board, and Dr. W. S. Carter to be clinical lecturer on pediatrics. Dr. Thomas Flavin, whose failing health has caused him to resign the position of demonstrator of anatomy which he has for years so efficiently filled, was appointed emeritus demonstrator of anatomy. Dr. William Gammon was made associate in pathology; Dr. L. E. Magnenat, demonstrator of pathology; Dr. M. Charlotte Schaeffer, demonstrator of Normal Histology; Dr. Kenneth H. Aynesworth, demonstrator of anatomy; Dr. C. C. Jones, demonstrator of obstetrics and gynecology. The Regents also authorized the establishment of demonstratorships in physiology and in pharmacy. With the exception of these changes the teaching staff remains as heretofore.

